# On the Wegener granulomatosis associated region on chromosome 6p21.3

**DOI:** 10.1186/1471-2350-7-21

**Published:** 2006-03-09

**Authors:** Paweł Szyld, Peter Jagiello, Elena Csernok, Wolfgang L Gross, Joerg T Epplen

**Affiliations:** 1 Human Genetics, Ruhr-University, Bochum, Germany; 2 Clinical Molecular Biology, Christian-Albrechts-University Kiel, Germany; 3 Rheumatology, University Hospital Luebeck and Rheumaklinik Bad Bramstedt, Germany

## Abstract

**Background:**

Wegener granulomatosis (WG) belongs to the heterogeneous group of systemic vasculitides. The multifactorial pathophysiology of WG is supposedly caused by yet unknown environmental influence(s) on the basis of genetic predisposition. The presence of anti-neutrophil cytoplasmic antibodies (ANCA) in the plasma of patients and genetic involvement of the human leukocyte antigen system reflect an autoimmune background of the disease. Strong associations were revealed with WG by markers located in the major histocompatibility complex class II (MHC II) region in the vicinity of human leukocyte antigen (*HLA*)-*DPB1 *and the *retinoid X receptor B *(*RXRB*) loci. In order to define the involvement of the 6p21.3 region in WG in more detail this previous population-based association study was expanded here to the respective 3.6 megabase encompassing this region on chromosome 6. The *RXRB *gene was analysed as well as a splice-site variation of the *butyrophilin-like *(*BTNL2*) gene which is also located within the respective region. The latter polymorphism has been evaluated here as it appears as a HLA independent susceptibility factor in another granulomatous disorder, sarcoidosis.

**Methods:**

150–180 German WG patients and a corresponding cohort of healthy controls (n = 100–261) were used in a two-step study. A panel of 94 microsatellites was designed for the initial step using a DNA pooling approach. Markers with significantly differing allele frequencies between patient and control pools were individually genotyped. The *RXRB *gene was analysed for single strand conformation polymorphisms (SSCP) and restriction fragment length polymorphisms (RFLP). The splice-site polymorphism in the *BTNL2 *gene was also investigated by RFLP analysis.

**Results:**

A previously investigated microsatellite (#1.0.3.7, Santa Cruz genome browser (UCSC) May 2004 Freeze localisation: chr6:31257596-34999883), which was used as a positive control, remained associated throughout the whole two-step approach. Yet, no additional evidence for association of other microsatellite markers was found in the entire investigated region. Analysis of the *RXRB *gene located in the WG associated region revealed associations of two variations (rs10548957 p_allelic _= 0.02 and rs6531 p_allelic _= 5.20 × 10^-5^, OR = 1.88). Several alleles of markers located between *HLA-DPB1*, SNP rs6531 and microsatellite 1.0.3.7 showed linkage disequilibrium with *r*^*2 *^values exceeding 0.10. Significant differences were not demonstrable for the sarcoidosis associated splice-site variation (rs2076530 p_allelic _= 0.80) in our WG cohort.

**Conclusion:**

Since a microsatellite flanking the *RXRB *gene and two intragenic polymorphisms are associated significantly with WG on chromosome 6p21.3, further investigations should be focussed on extensive fine-mapping in this region by densely mapping with additional markers such as SNPs. This strategy may reveal even deeper insights into the genetic contributions of the respective region for the pathogenesis of WG.

## Background

Wegener granulomatosis (WG) is a granulomatous disorder belonging to the heterogeneous group of systemic vasculitides (SV). A common feature of SV is the inflammation of the endothelium [1,2]. SV are classified according to the size of affected vessels and the type of auto-antibodies, namely anti-neutrophil cytoplasmic antibodies (ANCAs), which are used for differential diagnosis [3,4].

WG has an annual incidence of 5–10/million individuals in Caucasians [5]. The pathophysiology of WG still remains largely unknown with a supposedly multifactorial basis [6,7]. Presence of ANCA in the plasma of ~90% of WG patients reflects autoimmune background of the disease. In WG patients ANCAs are mostly directed against proteinase 3 (PRTN3), presented in primary azurophil granules of polymorph nuclear neutrophils (PMN) and lysosomes of monocytes [8,9]. After cytokine priming of PMN, PRTN3 translocates to the cell surface where ANCAs can bind and activate PMN resulting in a respiratory burst and release of proteolytic enzymes [10]. This may then lead to a self sustaining inflammatory process.

Several candidate genes such as *PRTN3*, *α1-antitrypsine*, adhesion molecule CD18 or *interleukin 1 *and its receptor have been investigated for WG association [11-15]. In addition, there is genetic evidence that the human leukocyte antigen (HLA) system is involved in WG development [16-19]. Yet, mostly these studies showed exclusively spurious WG associations. Recently, an extended association screen (EAS) revealed strong WG association of a microsatellite marker (UCSC May 2004 Freeze chr6:31257596-34999883) located in major histocompatibility complex class II (MHCII) region in the immediate vicinity of the *HLA-DPB1 *and *retinoid X receptor β *(*RXRB*) genes in two German populations [20]. Genotyping 19 alleles of the *HLA-DPB1 *gene with increased frequency of the HLA-DPB1*0401 allele supported the evidence that this region harbours at least one genetic factor for WG. But further association mapping encompassing a region of ~280 kb with additional microsatellite and SNP markers did not allow to decide between several alternatives. Either this region harbours one major locus for WG, encompasses two susceptibility loci or the association of specific marker alleles is due to linkage with a causative locus at some distance. In this context, the complex linkage disequilibrium (LD) patterns are crucial with areas of densely packed recombination hot spots as well as large LD blocks on chromosome 6p thus further complicating to define associations in this region [21,22]. In a recent study on the granulomatous disorder sarcoidosis, a fine mapping approach using SNPs led to the definition of an HLA independent genetic factor, *BTNL2 *[23]. This study underlines the necessity of investigations on larger regions encompassing defined associations in order to evaluate aforementioned considerations on extended LD on chromosome 6, *i.e*. how far an association can extend, or HLA independent risk factors. Here, we expanded association mapping to a 3.6 mb region encompassing the WG associated 6p21.3 as well as adjacent regions with additional microsatellites. This study was carried out in a two-step population based design using pooled DNA for the initial scan and, subsequently individual genotyping of markers that differed statistically significantly between WG patients and controls.

Furthermore, we focussed on two candidate genes on chromosome 6, *RXRB *and *butyrophilin-like 2 *(*BTNL2*) located in the vicinity or even in the respective WG-associated region [20]. *RXRB *presents as a good candidate as the protein binds to hormone nuclear receptors such as the vitamin D receptor (VDR) [24]. VDR and its ligand calcitriol have widespread influence on immuno-regulatory systems [25-28]. Thereby, *RXRB *variation may be involved in the pathogenesis of WG. In order to find functionally relevant polymorphisms the entire coding region and ~593 bp of the promoter were screened for variations. Furthermore, a recently described splice-site polymorphism of the *BTNL2 *gene has been reported to represent a HLA independent risk factor for another granulomatous disorder affecting the lung, namely sarcoidosis [23]. *BTNL2 *is located on 6p21.3 in the vicinity of the *HLA-DRB1 *locus. On the basis of sharing traits in WG and sarcoidosis such as granulomas and pulmonary affections, the functionally relevant splice-site polymorphism was genotyped in our cohorts.

## Methods

### Patients

German WG patients were clinically diagnosed according to international standards by applying the 1990 classification criteria of the American College of Rheumatology [29] and the definitions of the 1994 Chapel Hill Consensus Conference [3]. All patients have defined ANCA-status (n_positive _= 151; n_negative _= 30). WG was biopsy-proven in the German reference centre for vasculitis (Department of Pathology, University of Schleswig-Holstein Campus Luebeck, Germany) by 2 different observers. Control groups comprised healthy adults from northern Germany and the Ruhr area (Germany). The institutional Ethics Committee of Bochum Ruhr-University approved this study. Informed consent was obtained from all individuals.

### Pooling of DNA

DNA pooling was performed as described before [20]. Briefly, DNA concentrations were measured spectrophotometrically in 4 steps, and samples were diluted accordingly. The final DNA concentration in pools was 50 ng/μl. In order to control for artefacts during pooling, 150 ANCA-positive patients were divided into 3 sub-pools and 100 controls from the northern German cohort into 2 sub-pools, respectively.

### Microsatellite marker panel

A panel of 94 markers was designed encompassing the WG associated 6p21.32 and flanking regions (annotated by Santa Cruz genome browser (UCSC): chro6:31255920-34938760, May 2004 freeze, see [30]). Microsatellites were chosen utilizing the simple-repeat option of UCSC. The average distance between microsatellite markers was 41 kb (standard deviation ~30 kb) with five gaps of distances ≥100 kb (<152 kb). Most of the microsatellites harboured dinucleotide repeats (68%), the remaining comprised tri-, tetra- or penta-nucleotide repeat motifs. Further information about the microsatellites is available as additional file 1 (website: [31]). No marker revealed significantly differing 'intra sub-pool' distributions as reflected by the p value (see below).

### PCR

For fragment analysis we used fluorescence 5'FAM labelled, tailed oligonucleotide added to the 5'-part of the sequence specific primer as described before [20]. Primers were designed with the Primer Express 2.0 Software (Applied Biosystems, Foster City, USA). Melting temperature was set to 55°C. Three primers were used for PCR: tailed forward primer (tailed F), reverse primer and labelled primer (labelled F) corresponding to the 5'-tail sequence of tailed F. Reaction mix consisted of 1 × PCR buffer (Qiagen GmbH, Hilden, Germany), 1.5 pmol labeled F, 0.2 mM each dNTP, 3 mM MgCl_2_, 0.2 pmol tailed F, 1.5 pmol reverse primer, 0.25 U Qiagen Hot Start Taq (Qiagen) and 50 ng DNA. PCR was performed in a Biometra T-Gradient Thermoblock (Biometra, Göttingen, Germany) using the following characteristics: activation step at 95°C for 15 min; 35 cycles of denaturation at 95°C for 1 min, annealing at 55°C for 1 min and extension at 72°C for 1 min; and a final extension at 72°C for 10 min.

### Initial screen

Electrophoresis and data analysis were performed on a 48-well ABI377 slab-gel system using ABI Genotyper software as described before [20]. Data analysis generated marker-specific allele image profiles (AIPs) representing distributions of alleles within each sub-pool.

### Statistical analysis

AIPs of WG pools were compared with those of the control cohort. Peak heights were cumulated to 100% (representing 100 alleles in each pool, respectively). Afterwards allele frequencies (corresponding to peaks height) were calculated according to the estimated total allele count. Association was tested for each marker by comparison of the AIPs from WG-DNA pools with those of controls by contingency tables using a significance level of p = 0.05. In order to focus the statistics on major effects, alleles with a frequency of less than 5% were summarized to one allele. Next, frequency distributions were compared by means of contingency tables as mentioned above. The resulting p values were corrected for multiple testing using QValue software [32,33] with a 'cut off' of 5%. Nevertheless, non-corrected p values were simply ranked according to their evidence for association in order to select markers for further studies. In addition, intra-population differences were tested by comparing WG pools with each other as well as both control pools.

### Individual genotyping

In order to exclude false positive significances, patients and controls were individually genotyped for respective markers. Genotyping was performed on the Beckman Coulter CEQ8000 8-capillary system using 'Fragment Analysis Module' software (Beckman Coulter, Inc., Fullerton, USA). Reaction mix contained 1 × PCR buffer (Qiagen), 0.75 pmol fluorescent labelled F, 0.2 mM each dNTP, 3 mM MgCl_2_, 0.2 pmol tailed F, 1.5 pmol reverse primer, 0.25 U Qiagen Hot Start Taq (Qiagen) and 50 ng DNA. Parameters for amplification were used as described above. Allele and genotype frequency of WG patients and controls were compared by χ^2^-testing. Additionally, Hardy-Weinberg equilibrium was tested using Genepop software [20].

### Screening of the coding and a 593 bp promoter region in the *RXRB *gene

The coding and promoter regions (593 bp) of the *RXRB *gene were initially screened by single strand conformation polymorphism (SSCP) with 48 patient (ANCA-positive) and 48 control DNAs. The *RXRB *sequence was downloaded from UCSC database (NM021976, July 2003 Freeze). Oligonucleotides were designed using Primer Express 2.0 Software (Applied Biosystems) with an optimal annealing temperature of 57°C (see table 1). PCR was performed using Qiagen HotStar Taq (Qiagen) with an initial denaturation at 95°C for 15 min, 35 cycles of 94°C for 30 sec -57°C for 30 sec -72°C for 30 sec and a final extension at 72°C for 10 min. PCR conditions were chosen as recommended by Qiagen. Fragments were labelled with α-dATP (exons 2–10; Hartmann Bioanalytics, Braunschweig, Germany) and α-dCTP (exon 1a and 1b; Hartmann Bioanalytics). SSCP were analysed on 6% and 5% polyacrylamide gels with either 5% or 10% glycerol. The gels were run at room temperature at 35 W. Amplificates containing obvious band shifts were sequenced using dye terminator cycle sequencing on an ABI377 automatic sequencer (Applied Biosystems). Statistical comparisons of allele frequencies and genotypes were based on the χ^2 ^test. In addition, Hardy-Weinberg equilibrium has been tested for all SNPs.

**Table 1:** Oligonucleotides and restriction enzymes used for SSCP analyses of the exonic and 593 bp promoter regions of the *RXRB *gene.

**Exon**	**Oligonucleotide sense/antisense**	**PCR product (bp)**	**Restriction enzyme; length of fragments (bp)**
**01a**	CGGTATCCCTACTCTCAGCCA/GCGGGATCCAGCCAGG	164	-
**01b**	GGTGCGAAAAGAAATGCA/CACTGGCTCGCCTGCC	202	-
**02**	CTCTTGTGTGTCTGTGTGCCT/GGTTAGAGGATTGGAAGGTCA	403	AvaII 176/227
**03**	GACTTCCCTATTTCCCCCAT/TCTCGGAGAAGAGGAGGCT	207	-
**04**	TGAAGGTGTCTCCATGCAAC/AGAAGGGCATGTGGTCTAAGA	275	BsmFI 126/149
**05**	CTGATGAGGCCGTAAGGATA/GTGGATTGACCCCAACACT	280	Eco24I 130/150
**06**	TCCATAACTCTTACCCCCGT/CTGGGTACGCAAGGTAAGG	255	MboII 102/153
**07**	CCTGATTTCTGGCTCCTGAC/CCTCTATCTACATGCCAGCCT	209	-
**08**	GCTGAGCTGTGACCTTTGAGT/TCTCGGAGAAGAGGAGGCT	199	-
**09**	CTCCTCTCTTCCCTGCCAT/CCTCATAGCACTCCCCACC	199	-
**10**	CTCAATCCCCTTCTCCCAC/AGTGTGAGAAGCACCACGTC	209	-
**Promoter (593 bp)**			
**Sys1**	GGGCTTAATTCGACCCAA/GCATTTCTTTTCGCACC	213	-
**Sys2**	GCTAACAGGCCGGAGGAGA/GGATTGATCGGAGGATTAGCT	212	-
**Sys3**	CTCTCCTTTCCCGGTTTG/TCGTCTAGTTGGAAACCGAG	234	-
**Sys4**	CTCTTTATCCCGAACCACCT/TCTCGCGGGATCTAAAGG	221	-
**Sys5**	CATCACGCTGACCAGAGG/TGGTGAAAGATTAGTGTCCCA	214	-

### Genotyping of rs6531

The sequence variation rs6531 was genotyped in 151 ANCA-positive patients and 201 controls by RFLP analysis. A restriction site for DdeI (NewEnglandBiolabs, Ipswich, USA) was generated by using the tailed mismatch oligonucleotide, mm*antisense*-*CATCGCTGATTCGCACAT*CATCAATGGATCGGTCTGA. PCR was performed by 'the tailed primer' method using three oligonulceotides: the mm*antisene*-primer, an oligonucleotide corresponding to the tail and the forward oligonucleotide *sense*-CCGATCTTTAGTGACCCCAGT. Restriction with DdeI results in a 226 bp fragment (T allele) and in case of the C allele in 2 fragments of 192 and 34 bp, respectively. Electrophoresis was done on a 3% agarose gel containing ethidium bromide. The results were analysed with the χ^2 ^test and Hardy-Weinberg equilibrium was controlled.

### Genotyping of the Val95Ala polymorphism in the *RXRB *gene

The previously reported the Val95Ala polymorphism [34] was genotyped by RFLP analysis in 94 patients and 90 controls. Fragments were amplified using oligonucleotides Val95Ala sense-CGGTGGGGTATTAGAGAATT and Val95Ala antisense-CCCATGGAAGAACTGATGACTGG. PCR was performed with Qiagen HotStar Taq (Qiagen) as described above with an annealing temperature of 55–60°C and different MgCl_2_-concentrations generating a 300bp fragment. The 2 alleles (Val, T allele; Ala, C allele) are detected as 232 bp and 193 bp bands, respectively.

Electrophoresis was done in ethidium bromide containing 2% agarose gels. In order to control the results of the RFLP analysis, DNAs of 15 patients and 20 controls were sequenced as described above. Association was verified with the χ^2 ^test and Hardy-Weinberg equilibrium.

### Genotyping the functionally relevant *BTNL2 *variation rs2076530

Primer extension analysis was used to genotype the truncating splice site mutation in 180 patients and 261 controls. Two primers were designed for amplification, forward TCCAGATACTCAGTGCCAGA, reverse: TTGTCCAGGAACTAGCATATT. PCR conditions were: 1 × PCR buffer (Qiagen), 0.2 mM each dNTP, 0.2 pmol forward primer, 0.2 pmol reverse primer, 0.25 U Qiagen Hot Start Taq (Qiagen) and 50 ng DNA. The reaction was performed with initial denaturation at 95°C for 15 min, 35 cycles of 94°C for 30 sec -60°C for 30 sec -72°C for 30 sec and a final extension at 72°C for 10 min The product was purified with magnetic beads system (AMPure; ABgene, Epsom, UK). A primer extension reaction was performed with the interrogation primer: GCCCAGTTTGGATCTGAAGGTGGTA using Beckmann CEQ™ SNP-Primer Extension Kit (Beckman Coulter) under recommended conditions. Electrophoresis and data analysis was performed using Beckman Coulter CEQ8000. Statistical analysis was based on the χ^2 ^tests, and allele frequencies were tested for Hardy-Weinberg equilibrium.

### LD analyses

We calculated pair-wise LD between *HLA-DPB1 *alleles (genotypes obtained from a previous study [20], see additional file 2, website: [35]), rs6531 and the significantly associated microsatellite #1.0.3.7 in our control and patient cohorts. Microsatellite, SNP and *HLA-DPB1 *alleles with frequencies below 0.05 were excluded. For pairwise LD calculation we presumed each microsatellite allele to be biallelic, *i.e*. one distinct allele against all others pooled. The same procedure was applied for the most frequent *HLA-DPB1 *alleles *0401, *0402, *0201 and *0301. The strength of LD between all three markers was quantified by D' and r^2 ^values. LD analyses were calculated by Haploview 3.2 [36].

## Results

### Initial association mapping

**Figure 1:**
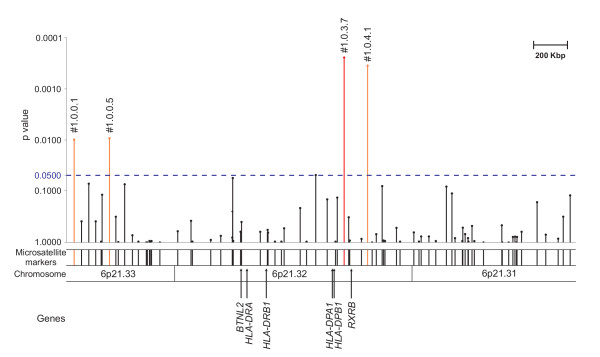
**Map of the investigated *HLA *region with p values of the markers used in the initial screen**. Figure shows the investigated fragment of chromosome 6 comprising the previously defined WG associated region encompassing the *HLA-DPB1 *and *RXRB *genes. Vertical lines represent studied microsatellites. The lengths/heights of these lines correspond to p values after analyses in the initial screen with pooled DNA. The 4 markers, which were genotyped individually, are labelled. The p values of markers #1.0.0.1, #1.0.0.5 and #1.0.4.1 (pool analysis) could not be replicated by genotyping individually. Drawn in red is the marker (#1.0.3.7) that remained significantly different after individual genotyping. This marker has been used as a positive control (see text). For further information of fine localisation of each investigated marker and inter-marker distances see additional file 1.

Eighty four markers were analysed in an initial scan (after exclusion of non-polymorphic and recurrently artefact-producing microsatellites). Thereof, five microsatellites revealed significant differences in allele distribution between WG patients and controls (see table 2). Correction for multiple comparisons with QValue rejected significance for all results. Nonetheless, markers were simply ranked according to their p values. Thereafter, four markers were selected for individual genotyping (see figure 1). One microsatellite showed only marginal significance (#1.0.3.4) after summation of rare alleles (<5%) and thus was excluded from further investigations. Individual genotyping confirmed exclusively one marker (adjacent to *RXRB*), the positive control, as statistically significantly different: #1.0.3.7 (p = 3 × 10^-4^, see table 2).

**Table 2:** P values for microsatellite markers after the initial step and individual genotyping.

		**p values**	
**Code of marker**	**Number of alleles**	**after analysis with pooled DNA**	**after summation of rare alleles (<5%)**

#1.0.0.1	5	0.02	0.01
#1.0.0.5	12	0.03	0.01
#1.0.3.4	3	0.04	0.05
#1.0.3.7*	5	2 × 10^-4^	3 × 10^-4^
#1.0.4.1	6	1 × 10^-4^	4 × 10^-4^
			

	**p values**		

**Code of marker**	**after analyses of each single allele (most significant allele)**	**after individual genotyping (p_c _value)**	**after correction by Q-value**

#1.0.0.1	0.03	n.s.	n.s.
#1.0.0.5	>0.05	n.s.	n.s.
#1.0.3.4	0.03	n.d.	n.d.
#1.0.3.7*	< 1 × 10^-4^	4 × 10^-5 ^(p_c _= 3 × 10^-4^; c = 5)	n.s.
#1.0.4.1	3 × 10^-3^	n.s.	n.s.

n.s. = not significant; n.d. = not determined; * = positive control

### Analyses of the *RXRB *gene

SSCP analyses in the *RXRB *gene did not reveal any variations in exons 1–5, 8–10 and the 593 bp promoter region. Similarly, the Val95Ala polymorphism was neither detectable by RFLP nor by sequence analyses in our patient and control cohorts. Yet, the analysis of the PCR product comprising exon 6 revealed a band shift. Subsequent sequencing showed an intronic *CT*-INDEL polymorphism which has been reported before (rs10548957). Statistical analysis of the allele frequencies revealed a p value of 0.02 with a minor allele frequency of 0.07 in the control cohort (n = 47) and total absence in the patients (n = 43). In exon 7 a single nucleotide polymorphism (SNP) (rs6531) was detected using SSCP and sequencing. Comparison of allele frequencies of patients (n = 151) *vs*. controls (n = 201) revealed significant p values (see table 3 for detailed analyses). Additionally 28 ANCA-negative patients were genotyped for this SNP and compared to either the ANCA-positive patients or the control cohort. There is a significant deviation between the ANCA-negative and -positive patients regarding this SNP. The ANCA-negative cohort did not differ from the control group.

**Table 3:** Genotyping of rs6531 in the *RXRB *gene.

**Genotypes/phenotypes/alleles**	**Patients ANCA^+ ^(n = 151)**	**Patients ANCA^- ^(n = 36)**	**Controls (n = 201)**	
	
**CC**	36	15	92	
**CT**	87	19	88	
**TT**	28	2	21	
				
**CC+CT (*vs*. TT)**	123	34	180	
**TT+CT (*vs*. CC)**	115	21	109	
				
**C**	159	49	272	
**T**	143	23	130	
				
	**ANCA^+ ^against ANCA^- ^patients**		**ANCA^+ ^patients against controls**	

**Genotypes/phenotypes/alleles**				

	**P value**	**OR (95 % C.I.)**	**P value**	**OR (95 % C.I.)**

**CC**	0.03		2.3 × 10^-5^	
**CT**	0.10	1.91 (0.87 to 4.16)*	1.6 × 10^-4^	2.53 (1.55 to 4.11)*
**TT**	0.02	5.83 (1.23 to 27.65)*	0.03	5.89 (3.01 to 11.53)*
				
**CC+CT (*vs*. TT)**	0.07	0.26 (0.06 to 1.14)	0.03	0.51 (0.29 to 0.94)
**TT+CT (*vs*. CC)**	0.03	2.26 (1.17 to 4.88)	2.3 × 10^-5^	2.70 (1.69 to 4.30)
				
**C**	1.0 × 10^-2^		5.2 × 10^-5^	
**T**		1.91 (1.11 to 3.30)		1.88 (1.38 to 2.56)

OR = odds ratio, C.I. = confidence interval.

*Odds ratios for the CT and TT genotypes are contrasted against the CC genotype. No evidence for statistical difference between the ANCA-negative WG patients and the control cohort was revealed, when calculating all off above-used comparisons.

### LD analyses

**Figure 2:**
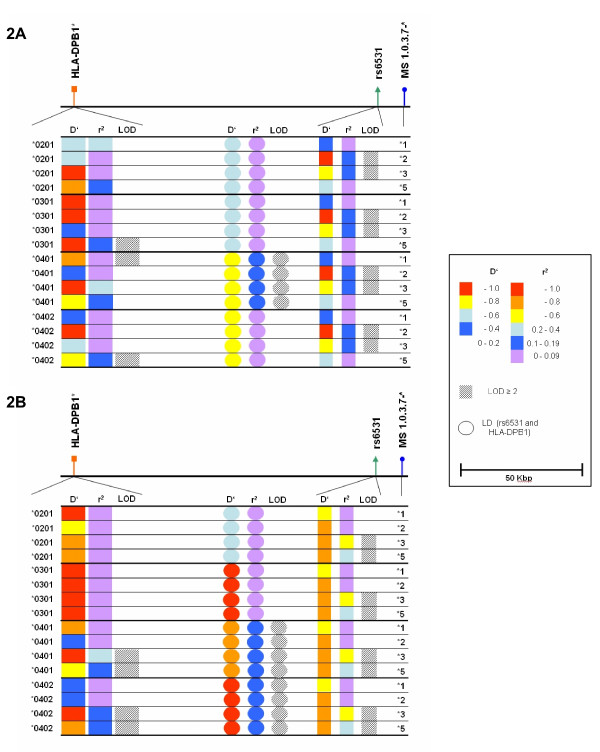
**Graphical overview of LD analyses with frequent *HLA-DPB1 *alleles, the rs6531 polymorphism in the *RXRB *gene and alleles of the significantly WG-associated #1.0.3.7 microsatellite marker**. Pair-wise LD evaluated by *D' *and *r*^*2 *^between markers in the previously found WG associated chromosome 6 region for controls (2A) and patients (2B). Vertical lines with circle, triangle and square represent the relative localisation of each tested marker (*HLA-DPB1*, *RXRB *rs6531 and microsatellite marker #1.0.3.7, respectively). *D' *and *r^2 ^*values are indicated by colour coding. Logarithm of odds (LOD) values equal to or exceeding 2 are represented by dashed rectangles, circles or squares. Rectangles are related to LDs between the microsatellite #1.0.3.7 and *HLA-DPB1 *alleles. Circles represent LD values between *HLA-DPB1 *and the SNP. Squares show LDs between the microsatellite alleles and rs6531 SNP. The investigated alleles of the microsatellite markers are shown at the left hand side of the figure, whereas the different designations of the *HLA-DPB1 *alleles are depicted at the right hand side. MS: microsatellite.

Several alleles between *HLA-DPB1*, rs6531 and microsatellite marker #1.0.3.7 showed LD with *r*^*2 *^values exceeding 0.10 (for detailed description see figure 2A and 2B). For example, strong LD exists between HLA-DPB1*0401 and microsatellite allele *3 as well as the rs6531 polymorphism in the patient and control cohorts.

### Analysis of the functionally relevant *BTNL2 *variation

Genotyping of rs2076530 polymorphism in 180 patients and 261 controls (Northern Germany and Ruhr area populations) did not reveal any statistically significant difference in allele, phenotype and genotype frequencies between all WG patients and healthy individuals (see table 4). In addition, when dividing the patient cohort into ANCA^+ ^and ANCA^-^patients, significant differences were not detected between the respective patient and control cohorts (data not shown).

**Table 4:** Genotyping of rs2076530 in the *BNTL2 *gene.

**Genotypes/phenotypes/alleles**	Patients ^*1 ^(n = 180)	**Controls (n = 261)**	**P value**
**GG**	68	79	0.27
**GA**	76	135	0.14
**AA**	36	47	0.94
			
**GG+GA**	144	214	0.84
**AA+GA**	112	182	0.19
			
**G**	212	293	0.80
**A**	148	229	

^*1 ^ANCA positive (n = 150) and ANCA negative (n = 30) patients. No statistically significant difference between the ANCA positive and negative patients was evident.

## Discussion

The approach using pooled DNA and allele image profiles for microsatellite based association studies was used to investigate groups of candidate genes for different multifactorial disorders revealing novel predisposition factors for respective diseases [20,37]. For these studies markers were chosen located intra- or juxtagenically to distinct genes. Here, we used a population based association study approach with microsatellites and DNA pooling for scanning a ~3.6 mb region on 6p21.3. This chromosomal segment includes the previously characterized, WG associated region [20]. Several other studies have revealed associations of *HLA *genes with WG and other vasculitides (*e.g*. [17,19,38-41]). Yet, most of these results could not be replicated. Considering the highly variable *HLA *complex, the strong positive selection effects on this region in combination with the relatively limited number of investigated patients, definitive conclusions are truly challenging. By using our EAS approach we searched for further loci for WG or whether the identified *HLA-DPB1/RXRB *associations are due to linkage with other factors located more distantly on 6p21.3 comprised in the genomic region investigated here. Yet, we could neither reveal any evidence for an additional locus nor for an expanded, significantly associated '*HLA-DPB1/RXRB*' region, respectively. In our study, the only marker (#1.0.3.7) which remained significantly associated has been reported before [20]. This marker is located in the immediate vicinity of the *RXRB *gene and ~125 kb centromeric to *HLA-DPB1*. Hence we calculated LD of the microsatellite, *HLA-DPB1 *alleles (with data from a previous study [20]; see additional file 2) and the significantly differing, intragenic rs6531 *RXRB *SNP. These analyses *e.g*. confirmed results of Rajisbaum et al. [42] which revealed significant LD between polymorphisms in *RXRB *and *HLA-DPB1*. In order to definitively investigate this very locus, a more detailed approach with a dense map of additional markers (preferential SNPs) appears mandatory.

It remains possible, that loci of small effects escaped detection in our microsatellite-based association mapping – a procedure known to ascertain strong associations. Such deficiency might be due to artefacts in the pooling procedure and analyses with pooled DNA such as length-dependent amplification of short microsatellite marker alleles or even the presence of null-alleles. In addition, the slab-gel system approach might reflect a further hindrance in this subtle procedure, since it is not 100% consistent. Altogether such shortcomings could result in false allele frequencies thus ignoring truly positive low-effect loci, for which extremely precise data are necessary. A further explanation for the negative results for most markers is that our panel may not represent the whole region completely. Hence the possibility remains that we missed causative alleles due to lack of LD between a distinct marker allele and the respective susceptibility factor. More detailed information on haplotype block structures and future definition of LD between multiallelic microsatellite markers may facilitate even more far-fetched interpretations of our analyses. Nevertheless, our data indicate that the here investigated region might harbour only one (major) locus associated with WG as represented by the control marker in the vicinity of *RXRB *(~5 kb) showing a positive result. By applying QValue correction tests, the significance of this marker would have been rejected. Similar controversies were presented recently in a study for narcolepsy utilizing EAS using correction according to Bonferroni [37]. Hence, further analyses on this region were performed regardless of the correction for multiple comparisons.

In addition to the indirect association study with microsatellites, we screened the entire coding (including the splice-sites) and the promoter region of *RXRB *in order to seek putative functionally relevant variations. In these analyses two polymorphisms were evident: an intronic *CT*-INDEL (rs10548957) variation revealing only a marginal association with WG, whereas the synonymous SNP (rs6531) in exon 7 was strongly associated (table 3). Nevertheless, this latter SNP does not affect known functional sequences influencing splicing and or regulation of gene expression. Therefore, the functional consequences of both polymorphisms are not immediately apparent. Both variations, however, confirmed the previously defined predisposing and protective haplotypes [20]. In this study only part of the potential promoter region was investigated. Therefore, the expression of *RXRB *may be influenced by polymorphisms/mutations in more distant promoter regions. Interestingly, the SNP in exon 7 might reflect differences between ANCA-negative and -positive patients. Whereas this polymorphism is associated in the ANCA-positive WG group, there is no statistical difference between the controls and ANCA-negative patient group. Although the amount of ANCA-negative patients in our study is rather small and therefore the statistics are almost underpowered, this result gives a further hint that both phenotypes may differ in their genetic background confirming recent studies, *e.g*. on the *intracellular tyrosine phosphates *(*PTPN22*) gene [43].

Although our initial mapping approach did not reveal an association in further regions in the *HLA *complex, we genotyped the previously found functionally relevant variation in the *BTNL2 *gene, which is associated with sarcoidosis and may present a predisposing/protective variation with low effect size [23]. Recently, several studies proposed common genetic factors for diseases characterised by autoimmune phenomena such as polymorphisms in the aforementioned *PTPN22 *gene or the cytotoxic T lymphocyte associated protein (CTLA4; see [44,45]). Considering common features of sarcoidosis and WG (*e.g*. autoimmune background, granulomata in the lung), variations within *BTNL2 *may represent risk factors for WG. Yet, in our study we did not find evidence for involvement of *BTNL2 *in the pathogenesis of WG, neither in the initial screen (both microsatellites flanking *BTNL2 *at ~25 kb: #1.0.2.5 and #1.0.2.6 showing p = 0.95 and p = 0.63, respectively) nor in individual genotyping with the functional rs2076530 polymorphism reported before (p = 0.42, table 4). As a remote possibility, different variations (of low effect size) in the *BTNL2 *gene may have predisposing/protective nature in the pathogenesis of WG due to allelic heterogeneity.

## Conclusion

Based on our largely negative association data in context with the defined WG associated region on 6p21.3, we submit that only a single region of ~280 kb exists that is responsible for WG predisposition in the *HLA *complex. In case several loci of low effect size would exist, novel alternative methodological approaches are necessary to validate their association with WG on chromosome 6.

## Competing interests

The author(s) declare that they have no competing interests.

## Authors' contributions

PS carried out the practical laboratory work, performed primary data analyses and drafted the manuscript. PJ initiated this study, prepared the initial version of the manuscript and performed statistical analyses. EC and WLG were responsible for the clinical characterisation of the entire patient cohort. JTE encouraged the critical approach, rewrote and finalized the paper. All authors read and approved the final manuscript.

## Pre-publication history

The pre-publication history for this paper can be accessed here:



## Supplementary Material

Additional File 1 Microsatellite marker panel, additional data. Click here for file

Additional File 2 *HLA-DPB1 *and microsatellite #1.0.3.7 genotyping information. Click here for file
