# Supplementary material for: On the Wegener granulomatosis associated region on chromosome 6p21.3
Source: BMC Med Genet. 2006 Mar 9;7:21. doi: 10.1186/1471-2350-7-21 (PMC1431512; doi:10.1186/1471-2350-7-21)
Supplement: Additional File 1 — Microsatellite marker panel, additional data. [file 1471-2350-7-21-S1.pdf]

Information on the microsatellite panel used for EAS

Pawel Szyld, Peter Jagiello *et al*., 2005

Legend

1) CoM = code of marker
2) chromStart corresponding to the UCSC freeze May 2004
3) chromEnd corresponding to the UCSC freeze May 2004
4) dist = Distance to previous marker
5) name = repetitive sequence
6) count = number of repetitions recommended
7) the tail CATCGCTGATTCGCACAT was added to the 5´-end of each forward Primer
8) reverse primer in 5' to 3' direction
9) length of PCR amplicon includes 18 bp tail

CoM	chromStart	chromEnd	dist	name	count	forwardPrimer	reversePrimer	PCR(length)
#1.0.0.0	31257596	31257623	0	AT	17,5	CATCGCTGATTCGCACATGCCACCATACCCCACTAATT	CCGTCAAACCTTGACTGTTC	189
#1.0.0.1	31312162	31312237	54539	TTTC	10,0	CATCGCTGATTCGCACATCCCCACAAAAAACCCCTGT	CACTCCAGACGCGGCAAC	259
#1.0.0.2	31367133	31367187	54896	GT	21,0	CATCGCTGATTCGCACATCAAGCATATCTGCCATTTGG	ATCTACCAGGGCCCAGTAGT	223
#1.0.0.2.1	31417350	31417376	50163	TTTG	8,8	CATCGCTGATTCGCACATTCCCTTAATTTTCTTTTTGCC	ACCATTGCACTCCAGCCT	195
#1.0.0.3	31457383	31457414	40007	GT	23,0	CATCGCTGATTCGCACATACCACAGTCTCTATCAGTCCA	TTTCTGCGTTTTCAGCCT	168
#1.0.0.4	31465639	31465733	8225	TC	15,5	CATCGCTGATTCGCACATCTGGCCTGGACCTACTGTATC	AAGGGTGTCTGACACCTGA	248
#1.0.0.5	31518185	31518230	52452	GT	11,0	CATCGCTGATTCGCACATGTCAGGGAGGAGAGAAAAGTG	GCTGGACTCTGCTAGGACA	220
#1.0.0.6	31562618	31562648	44388	TC	16,5	CATCGCTGATTCGCACATCTGGCCTGGACCTACTGTATC	GCAAGAAAGGGTGTCTGAC	190
#1.0.0.7	31579706	31579743	17058	TG	23,0	CATCGCTGATTCGCACATAGTGTTTTCCATTGCAGGC	CAAATCCAAACCACAATGAG	208
#1.0.0.7.1	31628000	31628046	48257	TTTTG	8,6	CATCGCTGATTCGCACATACCTCAAGTGATCCACCCA	TGGGCGACAGAATGAGACT	173
#1.0.0.8	31685399	31685441	57353	GT	14,0	CATCGCTGATTCGCACATGGATAACAGAACGAGACCCTG	TCAGTTGTGGTTGAAAAGAG	218
#1.0.0.9	31728077	31728116	42636	AC	18,0	CATCGCTGATTCGCACATCTTCCCACGGTGTTCCAG	GAGACCGGAAGTGACGGT	155
#1.0.1.0	31787584	31787634	59468	AC	20,0	CATCGCTGATTCGCACATCCAAGCAGCTACAGTTTGCT	GAACTCAGGATGGAGGGCT	186
#1.0.1.1	31791707	31791746	4073	GT	18,0	CATCGCTGATTCGCACATAGTTAAGGACCAGGGAGG	AGAAAAACAGAACTGGCACA	273
#1.0.1.2	31811564	31811606	19818	CA	21,5	CATCGCTGATTCGCACATTGCTCTCACTCTCAGGCCT	CAGCAGGGTTTAGAAGGGA	202
#1.0.1.3	31814868	31814906	3262	AC	22,5	CATCGCTGATTCGCACATCTTTCTCCCTCATAACCCCA	GGACTCAAAGCGGTGAACA	148
#1.0.1.4	31821837	31821870	6931	TG	22,0	CATCGCTGATTCGCACATTGGCCAGTTGTGTCCAGT	AGGCAGAGGTTGCATTGAG	152
#1.0.1.5	31885313	31885351	63443	AC	21,5	CATCGCTGATTCGCACATTGACAGGGCGAGACTGTCT	GGAGAGTTCATGGAGAGAG	185
#1.0.1.5.1	31949675	31949703	64324	ATTT	9	CATCGCTGATTCGCACATCACTTACTGCCATCCTTGCA	CCTGGGTGACAGAGCAAGA	133
#1.0.1.5.2	32014195	32014255	64492	TA	33,5	CATCGCTGATTCGCACATGGTGCCTGGAATGTAGAACAT	CGAGTTCGCACCACTGTAGT	183
#1.0.1.6	32142832	32142941	128577	AAGA	16,5	CATCGCTGATTCGCACATTACACTCTAGCCCAGGCGA	CCACCCACTTCCTCCACTA	217
#1.0.1.7	32153778	32153908	10837	AGAA	18,3	CATCGCTGATTCGCACATCCTGGGCAACAGAGCTCA	CACCACGCCTGACCACAA	210
#1.0.1.7.1	32211745	32211783	57837	AAAAC	7,8	CATCGCTGATTCGCACATGGTTGCAGTGAGCCAAGAT	CTCCTACCTTGTGCTGCTTC	179
#1.0.1.8	32287350	32287399	75567	AAAT	12,5	CATCGCTGATTCGCACATCAGACTGGGACTCCATCTCA	TGTTAGTGGTCCCCAAGGTA	167
#1.0.1.9	32352070	32352121	64671	AT	27,0	CATCGCTGATTCGCACATAGGTTCATCCATGTTGTCACA	GGCAAAGTCAAGAACTTTCC	237
#1.0.2.0	32362237	32362251	10116	TA	13,5	CATCGCTGATTCGCACATGAACATATGAACGCACGTGTC	CTGTGGAAAGCAGTTTGGAT	168
#1.0.2.1	32377724	32377750	15473	AAT	14,3	CATCGCTGATTCGCACATGGACATGCTATTTTTGCATGT	CACAACTACTCAACCCTGCT	262
#1.0.2.2	32442808	32442833	65058	GT	12,0	CATCGCTGATTCGCACATTCTTTCCTCAACTCTCAGGGA	ACCTCTCATTCTTGCTGGAA	178
#1.0.2.3	32442999	32443033	166	AG	26,5	CATCGCTGATTCGCACATCTTCCAGCAAGAATGAGAGGT	AGTATCCACTCACCAGGGCT	201
#1.0.2.4	32446163	32446199	3130	TA	10,5	CATCGCTGATTCGCACATAGGGAATCAGTGGTCTACGAA	TGTTTTGAGTATGCATTGCC	201
#1.0.2.5	32449996	32450040	3797	TG	14,5	CATCGCTGATTCGCACATGCTAGTCTGTGCCAAGGAACT	GAAGAAGGTGACCTGGACC	164
#1.0.2.6	32505535	32505566	55495	AC	12,5	CATCGCTGATTCGCACATCAGCTCAAGGACAAGAGAGGA	CCACATCCCAAAGACATGC	187
#1.0.2.7	32511353	32511396	5787	AC	16,0	CATCGCTGATTCGCACATCCTCCTTCAACACACAGACAC	TGGAATCTCATCAAGGTCAG	125
#1.0.2.7.2	32663146	32663179	151750	TAAA		CATCGCTGATTCGCACATCAACAAAGCGAGACTCCATC	GGAAATGGAGAGGCAGAAG	195
#1.0.2.8	32721614	32721644	58435	GT	16,0	CATCGCTGATTCGCACATCATTTTGGAGTTGTGTGCATT	GACTGAGTCTGAGTTGGGC	157

CoM	chromStart	chromEnd	dist	name	count	forwardPrimer	reversePrimer	PCR(length)
#1.0.2.9	32722622	32722639	978	AG	12,5	CATCGCTGATTCGCACATCAGGGATGAAACCAACAAGAT	CAGTCGGTGTCAATTTTTCTC	227
#1.0.3.0	32778135	32778163	55496	GT	11,5	CATCGCTGATTCGCACATTGCCCTGAGGTCTATGCTG	CAGCTCTTCTTAACCTGCCA	161
#1.0.3.1	32825101	32825141	46938	TG	10,0	CATCGCTGATTCGCACATTCAATGATTCATGAGCCAAGA	TGCCATTCAATGTAAGCCTTA	119
#1.0.3.1.2	32949071	32949113	123930	TATT	8	CATCGCTGATTCGCACATCCACCATGCCTGGCTAAT	GTTTGAGACCAGCCTGATCA	123
#1.0.3.2	32961966	32962013	12853	GATA	12,0	CATCGCTGATTCGCACATGGAGTCCATTGTGGTTGGA	TCATGGTTGCTTTTCCTTCA	207
#1.0.3.3	33008385	33008409	46372	CA	11,5	CATCGCTGATTCGCACATTCTTTCTCCCACTCCACTTCT	ATGGATGCTGCATGAGGA	207
#1.0.3.4	33074791	33074851	66382	TA	25,5	CATCGCTGATTCGCACATGGCAACATCTCAAGTCCTCTC	CTGCACAATGGAGTAATGCA	213
#1.0.3.4.2	33160605	33160661	85754	AAGG	14,2	CATCGCTGATTCGCACATAGAGCGAGATTATGTCTCAA	GGCTGGACTAGGAGCGT	149
#1.0.3.5	33218303	33218333	57642	AT	15,5	CATCGCTGATTCGCACATGAGTTTGAGACCAGCCTAGGA	TCCTGGGTAACTGGGACTAC	149
#1.0.3.6	33232070	33232096	13737	AC	13,5	CATCGCTGATTCGCACATCCATCAGCTTTATTTGGATCA	GCCCTCCTTGTGTCTTTGTTA	218
#1.0.3.7	33281727	33281753	49631	AG	13,5	CATCGCTGATTCGCACATGGTGGAGGGCAGAATCATT	AATGTTTGCCAGGAACTGTG	119
#1.0.3.8	33316475	33316533	34722	TG	17,5	CATCGCTGATTCGCACATAAGGAAGTCTCAAAGGCTCTG	TTGGTATGCCACTGCACAC	140
#1.0.3.9	33321669	33321709	5136	CA	20,5	CATCGCTGATTCGCACATTGGGCAGGACAAAGTATTTTC	GCCACTGGAGAAGAATCAAG	142
#1.0.4.0	33410343	33410371	88634	AC	15,5	CATCGCTGATTCGCACATCAGAGCAAGATTCCGTCTCA	GGCAGGGTTGGTTCTTTCT	157
#1.0.4.1	33453896	33453965	43525	AAAT	12,5	CATCGCTGATTCGCACATAGAGCGAGACTCCGTCTCA	GCACAGTGATCTCGTCAGTG	173
#1.0.4.2	33485461	33485509	31496	TG	24,5	CATCGCTGATTCGCACATGGAAGTGAAGACGGATCCA	GATAGCCCTGGGACATGGT	175
#1.0.4.3	33516845	33516900	31336	TG	13,5	CATCGCTGATTCGCACATGTCTCCTGAGTCCCCAGAGAT	CCTAGCCAAGAATGCAGAAG	149
#1.0.4.4	33558564	33558650	41664	TATC	11,5	CATCGCTGATTCGCACATTTGTTTGTCTTTTGGCTGTTG	AGGTTGCAGTGAGCCAAGT	201
#1.0.4.5	33570645	33570680	11995	GT	11,0	CATCGCTGATTCGCACATGCTAACAGCTCTGATCCACG	AGGCAGAGGTTGCAGTGAG	153
#1.0.4.6	33578171	33578205	7491	TG	13,5	CATCGCTGATTCGCACATTTATTCTCCTGCCTCAGCCT	TCCAGCATTGTGAAACCTCT	153
#1.0.4.6.1	33638415	33638451	60210	TTTA	9,2	CATCGCTGATTCGCACATACCTCTGCTTCCTGGGCT	TGAGCTGAGATCGCACCA	205
#1.0.4.7	33662781	33662810	24330	AC	15,0	CATCGCTGATTCGCACATCCCCTGAGTGAGTGCACA	CAGATGAGGAAACTGAGGCT	216
#1.0.4.8	33689516	33689545	26706	GT	15,0	CATCGCTGATTCGCACATGGTGTGCCAGTGTGTCTGA	ACAGCCAGGGAGAGTGTACA	169
#1.0.4.8.2	33790633	33790667	101088	TCAT	8,8	CATCGCTGATTCGCACATGACCTGTTTGGAATGTAGGCT	TGGGCCCCAGAAAATTATA	133
#1.0.4.9	33827239	33827274	36572	GT	18,0	CATCGCTGATTCGCACATGGGACAGACAACCTCTGCA	TGCCCCTAACCTGCTTCTA	157
#1.0.5.0	33842742	33842787	15468	CA	14,5	CATCGCTGATTCGCACATCCCCCAAACTTAGTAGCTTCA	AACTTAGGTGCAGGCCGTA	146
#1.0.5.1	33901448	33901504	58661	ATA	13,7	CATCGCTGATTCGCACATGAGACATTTTTGCGGCAAT	CTTCTGGGAATCATCCTGC	171
#1.0.5.2	33950685	33950725	49181	GT	18,0	CATCGCTGATTCGCACATATGTGTGGGCATGTGAGTG	GTGTTTCTAACCAGGACTGC	126
#1.0.5.3	34026337	34026378	75612	GT	16,5	CATCGCTGATTCGCACATAGTCTGTGGGTCTGTATGCCT	GAACTTCCCTGGGTGAGGT	129
#1.0.5.4	34065262	34065304	38884	TC	12,0	CATCGCTGATTCGCACATCTATGTGGCCATGGAAGTGT	ACTGCACTCTAGCCTGGGA	160
#1.0.5.5	34089739	34089759	24435	AC	10,5	CATCGCTGATTCGCACATTGCGGAGTAGCTGGGACTA	TGCGGAGTAGCTGGGACTA	142
#1.0.5.6	34107725	34107755	17966	AC	19,0	CATCGCTGATTCGCACATTGCGGAGTAGCTGGGACTA	GGCTTTGGAAGTCAGAAGGA	128
#1.0.5.7	34145403	34145443	37648	GT	20,5	CATCGCTGATTCGCACATACACACTCCCGGCTTTACA	CCAAATTCACTGGGTGCAT	154
#1.0.5.8	34163555	34163596	18112	AC	21,0	CATCGCTGATTCGCACATAGACATGCACCCCTACACAC	GCTAGCCTGGTTCTTAGGGT	154
#1.0.5.9	34173157	34173221	9561	TG	11,0	CATCGCTGATTCGCACATCAGTGCAAAGGCCATGTG	GCAGATCCAGGCACTAAGC	205

CoM	chromStart	chromEnd	dist	name	count	forwardPrimer	reversePrimer	PCR(length)
#1.0.6.0	34186865	34186909	13644	AC	20,0	CATCGCTGATTCGCACATCGTCTCATGCATTTGGATATC	AGTGTTCTTGCCTGTCTGACA	188
#1.0.6.1	34187144	34187179	235	CA	12,0	CATCGCTGATTCGCACATACACTCACAGGCCTCTTGGT	CACTGTTAATGCTGCCTCCA	129
#1.0.6.2	34212319	34212360	25140	AC	21,0	CATCGCTGATTCGCACATGCTTCCAGATCCTTCGTGA	GGTGTTTGCCTTTCTCCTCT	203
#1.0.6.3	34230346	34230383	17986	AC	19,0	CATCGCTGATTCGCACATACAGATGGGAGGTTAGATGGA	CTGCTTTTTGTCCCGCAT	137
#1.0.6.4	34256743	34256786	26360	AAT	14,7	CATCGCTGATTCGCACATGTGAGCCGAGATAGTGCCA	GACTTCAGTGATCTGCCTGC	204
#1.0.6.5	34296689	34296712	39903	CA	12,0	CATCGCTGATTCGCACATTGGTGCTTTTGCCAACTTA	AGCCTCCTGAGTAACTGGGA	195
#1.0.6.5.1	34372332	34372370	75620	ATAC	9,8	CATCGCTGATTCGCACATCCAGACCCTGTCGGTAAATAC	CATCGCTGGCACATATACTG	157
#1.0.6.6	34430150	34430191	57780	AC	17,5	CATCGCTGATTCGCACATCCTATGAACAGCCATTGCAC	GATTACAGGCGTGAGCCAC	146
#1.0.6.7	34434014	34434047	3823	AC	17,0	CATCGCTGATTCGCACATCCTCCAAGGTTTCTTACCCA	TGGGAAGTTTCAACATCCTGT	178
#1.0.6.8	34472071	34472095	38024	AC	25,0	CATCGCTGATTCGCACATGGACCCTGTCTCAAAACACAC	CACTGTCCCTGGCCTAGAAT	174
#1.0.6.9	34480476	34480525	8381	AC	12,0	CATCGCTGATTCGCACATTACTGATACATCCCCGGACA	CATGTGGAGAGGAGCAGGT	122
#1.0.7.0	34526036	34526070	45511	AAT	11,7	CATCGCTGATTCGCACATGCCAAGATCATGCCATTGT	GGCCTCTCAAAGTGTTGGA	161
#1.0.7.1	34538170	34538215	12100	GT	23,0	CATCGCTGATTCGCACATGGAAGCATGGAAGTTGTGTC	GCTTCCCTAAAGCTGAATTCA	187
#1.0.7.2	34539237	34539268	1022	CAT	10,7	CATCGCTGATTCGCACATTTGCAATAACCCTATGAGGC	ATGGCAACAGCACCTACCT	155
#1.0.7.2.1	34573794	34573834	34526	TGTT	10,2	CATCGCTGATTCGCACATGAATCACCTGGAGAACCTTGA	GACAGAGGGAGACCACGTCT	158
#1.0.7.2.2	34688972	34689003	115138	AAC	10.7	CATCGCTGATTCGCACATGGGTGACAGAGTGAGACCCT	TGGATGTGAGCTCCAACTTA	159
#1.0.7.3	34749583	34749613	60580	AC	16,0	CATCGCTGATTCGCACATGGGTGACAGAGAGATCCTGTC	CTCACCCCATTTCCTGTTCT	152
#1.0.7.3.1	34782160	34782193	32547	AAAAC	9	CATCGCTGATTCGCACATTGGCAACAGTGCAAGACTCT	GGAGTCTCTCTGTTGCCCAT	173
#1.0.7.3.2	34841161	34841209	58968	ATTT	11,8	CATCGCTGATTCGCACATTCCCTTTCTCCTTCCTGGA	GCAATGGAGCAAGACTCAAT	150
#1.0.7.3.3	34877452	34877491	36243	TTTA	9,8	CATCGCTGATTCGCACATGAAGCATTGTCCCTTGTCCT	GAGACAGAGCGAGACTCCG	208
#1.0.7.3.4	34928289	34928337	50798	CCTCT	9,8	CATCGCTGATTCGCACATGCAAGACAAAGTCCCCTGTAT	TGGGATAGCCAAAGGAATG	183
#1.0.7.4	34989375	34989406	61038	TTG	10,7	CATCGCTGATTCGCACATGTTACCATGCCTGGCCTAA	CGGAGGTTATGAGCCAAGAT	148
#1.0.7.5	34999846	34999883	10440	GT	19,0	CATCGCTGATTCGCACATATGGCCTCCCAGGATCTAT	GTCACAGACCTGGCTGGAT	167
