# Supplementary material for: On the Wegener granulomatosis associated region on chromosome 6p21.3
Source: BMC Med Genet. 2006 Mar 9;7:21. doi: 10.1186/1471-2350-7-21 (PMC1431512; doi:10.1186/1471-2350-7-21)
Supplement: Additional File 2 — HLA-DPB1 and microsatellite #1.0.3.7 genotyping information. [file 1471-2350-7-21-S2.pdf]

	Allele frequencies in patients (n=148)	Allele frequencies in controls (n=89)
HLA-DPB1*		
**0101*	0.01	0.05
**0201*	0.05	0.10
**0202*	0.00	0.01
**0301*	0.00	0.14
**0401*	0.77	0.47
**0402*	0.14	0.13
**0501*	0.00	0.01
**0601*	0.01	0.01
**0801*	0.00	0.00
**0901*	0.00	0.00
**1001*	0.00	0.03
**1101*	0.00	0.02
**1301*	0.00	0.00
**1401*	0.01	0.00
**1501*	0.00	0.01
**1601*	0.00	0.01
**1701*	0.00	0.01
**1801*	0.00	0.00
**1901*	0.00	0.01
	Total: 0.99	Total: 1.01

Frequencies and genotypes for LD analyses of *HLA-DPB1* alleles were adapted from Jagiello *et al*. (see reference 20).

	Allele frequencies in patients (n=146)	Allele frequencies in controls (n=78)
RXRB*		
**1*	0.03	0.14
**2*	0.07	0.13
**3*	0.47	0.37
**4*	0.01	0.01
**5*	0.42	0.33
	Total: 1.00	Total: 0.99

Frequencies of marker #1.0.3.7 alleles after individual genotyping
